# Persistent Rheb-induced mTORC1 activation in spinal cord neurons induces hypersensitivity in neuropathic pain

**DOI:** 10.1038/s41419-020-02966-0

**Published:** 2020-09-12

**Authors:** Xiaqing Ma, Wenjie Du, Wenying Wang, Limin Luo, Min Huang, Haiyan Wang, Raozhou Lin, Zhongping Li, Haibo Shi, Tifei Yuan, Wei Jiang, Paul F. Worley, Tao Xu

**Affiliations:** 1 Department of Anesthesiology, Shanghai Jiao Tong University Affiliated Sixth People’s Hospital, Shanghai, 200233 China; 2 The Solomon H. Snyder Department of Neuroscience, Johns Hopkins University School of Medicine, Baltimore, MD 21205 USA; 3 Shanghai Key Laboratory of Sleep Disordered Breathing, Affiliated Shanghai Sixth People’s Hospital, Shanghai Jiao Tong University, Shanghai, 200233 China; 4 Shanghai Key Laboratory of Psychotic Disorders, Shanghai Mental Health Center, Shanghai Jiao Tong University School of Medicine, Shanghai, 200030 China; 5 Department of Anesthesiology, Tongzhou People’s Hospital, Nantong, 226300 China

**Keywords:** Cellular neuroscience, Short-term potentiation

## Abstract

The small GTPase Ras homolog enriched in the brain (Rheb) can activate mammalian target of rapamycin (mTOR) and regulate the growth and cell cycle progression. We investigated the role of Rheb-mediated mTORC1 signaling in neuropathic pain. A chronic constriction injury (CCI) model was dopted. CCI induced obvious spinal Rheb expression and phosphorylation of mTOR, S6, and 4-E-BP1. Blocking mTORC1 signal with rapamycin alleviated the neuropathic pain and restored morphine efficacy in CCI model. Immunofluoresence showed a neuronal co-localization of CCI-induced Rheb and pS6. Rheb knockin mouse showed a similar behavioral phenotype as CCI. In spinal slice recording, CCI increased the firing frequency of neurons expressing HCN channels; inhibition of mTORC1 with rapamycin could reverse the increased spinal neuronal activity in neuropathic pain. Spinal Rheb is induced in neuropathic pain, which in turn active the mTORC1 signaling in CCI. Spinal Rheb-mTOR signal plays an important role in regulation of spinal sensitization in neuropathic pain, and targeting mTOR may give a new strategy for pain management.

## Introduction

Ras homolog enriched in the brain (Rheb) is an important regulator of growth and cell-cycle progression due to its critical roles in the activation of mTOR. Rheb localizes at the lysosome to activate mTORC1 and Rag7 proteins localize mTORC1 to the lysosome, allowing Rheb to activate the protein^1^. mTOR forms two distinct complexes, mTORC1 and mTORC2. While mTORC1 gates translation of most proteins by phosphorylated specific downstream effectors, like the eukaryotic initiation factor 4E-binding proteins (4E-BPs) and p70 ribosomal S6 protein kinases (S6Ks)^2^, and implicated in the regulation of cell growth, proliferation, and cell size.

Neuropathic pain is characterized by allodynia, hyperalgesia, and spontaneous pain^3^. Effective control of neuropathic pain is still inadequate^4^. Neuropathic pain related structural alterations in the central nervous system (CNS) is suggested as neuronal plasticity to induce such complex phenotypes^5^. Protein synthesis and regulation for the long-term memory formation is necessary for plasticity of neuronal synapses^6^. Adaptive changes in protein transcription and translation of neuroplasticity contribute to the development of neuropathic pain^7^. Rheb-induced mTORC1 activation may act as a mechanism and play a vital role in neuropathic pain-induced hyperalgesia and alodynia.

Neuroplasticity in the form of adaptive changes in protein transcription and translation may contribute to the development of chronic pain^7^. We observed an impaired morphine-induced antinociceptive effect in a specific Rheb knockin mouse which looks like the decreased efficacy of morphine in neuropathic pain. Here, we explored the Rheb-induced mTORC1 activation mediated increased spinal hypersensitivity in neuropathic pain, and to elucidate whether this Rheb-induced mTORC1 activation could be a candidate of the underlying mechanism of neuropathic pain.

## Results

### Expression of spinal Rheb was increased in neuropathic pain model

**Fig. 1:**
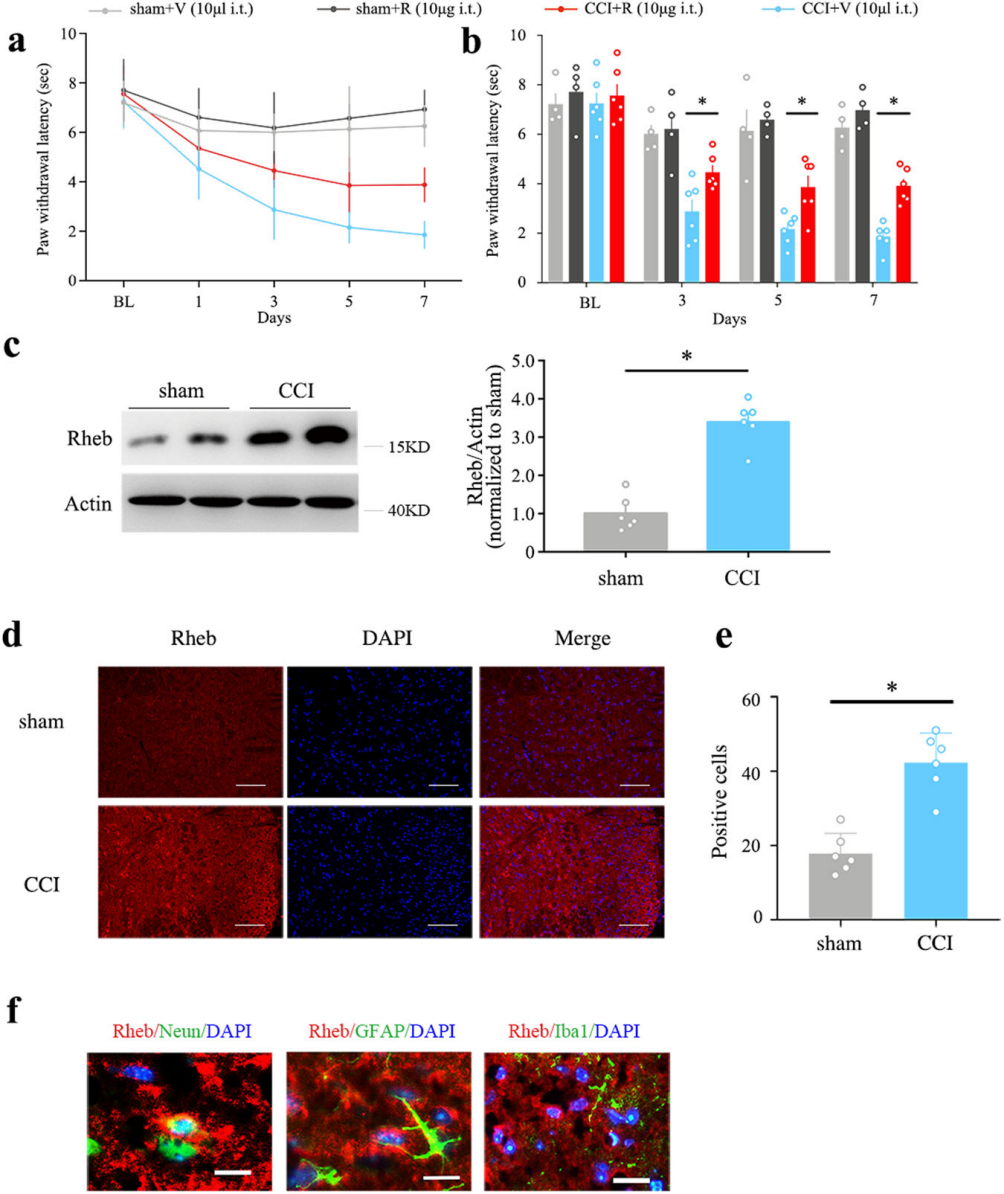
Neuropathic pain induces the expression of spinal Rheb. **a** Daily nociceptive behavior before CCI injury (BL) and on day 1, 3, 5, 7 after CCI injury with vehicle or rapamycin intrathecal injection once daily; **b** statistical analysis of nociceptive behavior of all these groups on baseline, day 3, 5 and 7 after CCI injury; **c** CCI significantly increase the expression of Rheb in spinal dorsal compared with sham group; **d, e** CCI significantly increase the immunofluorescence of Rheb in the spinal dorsal horn; **f** CCI-induced expression of Rheb mostly co-immunostaining with neuron (left, NeuN in green), but not astroglia (middli, GFAP in green), or microglia (right, Ibal in green). Repeated Measures Two-way ANOVA + Bonferroni (**b**), Student’s *t* test, two-tailed (**c**, **e**). *n* = 6 for all groups; *P < 0.05. Error bars are mean ± SEM. Overlaid points are individual subject.

The PWL showed a progressive decline during the 7 days course after the surgery, as compared with the sham group (Fig. 1a, b, *P* < 0.05). At the protein level, spinal Rheb was significantly increased in the dorsal horn in chronic constriction injury (CCI) group (Fig. 1c, *P* < 0.05 as compared with sham group). The immunofluorescence showed similar trends (Fig. 1d, e, *P* < 0.05 as compared with sham group). And CCI-induced expression of Rheb mostly co-immunostained with neuron (NeuN, green, Fig. 1f, left), but not astroglia (GFAP, green, Fig. 1f, middle), or microglia (Iba1, green, Fig. 1f, right).

### Upregulation of spinal Rheb and persistent activation of mTORC1 in spinal cord in neuropathic pain model

**Fig. 2:**
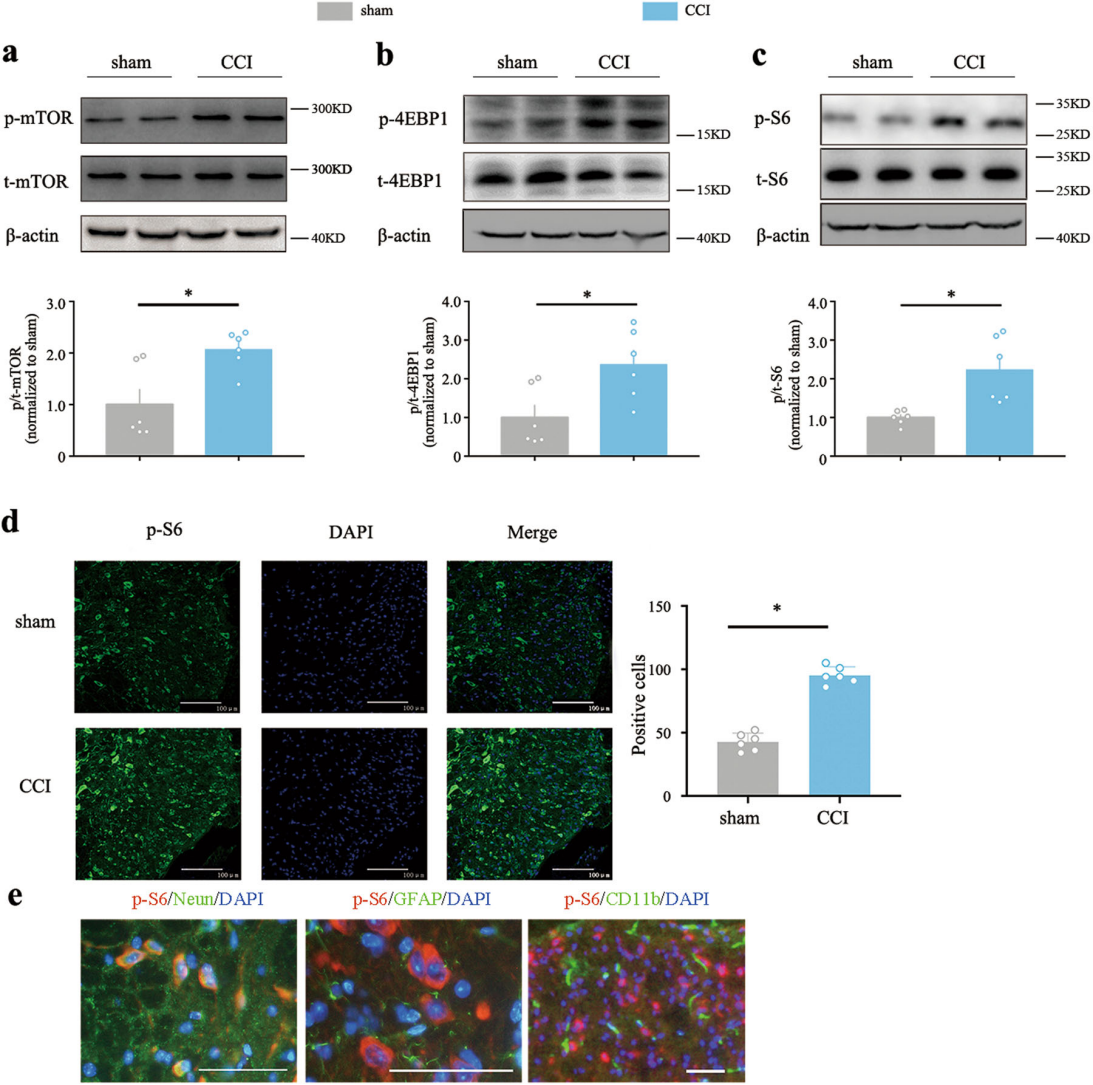
Rheb-induced activation of mTORC1 in Neuropathic pain and block the mTORC1 signal with rapamycin alleviate the development of neuropathic pain. **a, b, c** Western blot analysis of spinal cord to assess the activation of mTORC1 signal in sham mice and mice with CCI of the sciatic nerve after 7 days, CCI obviously increase the phosphorylation of spinal mTOR, S6 and 4-EBP1 (*n* = 6 for all groups); **d** CCI-induced phosphorylation of spinal S6 in the dorsal horn (*n* = 5); **e** CCI-induced phosphorylation of S6 co-immunostaining with neuron (left, NeuN in green), but nont astroglia (middle, GFAP in green), or microglia (right, CD11b in green) in the spinal dorsal horn, scale bars = 50 μm. Student’s *t* test (**a, b, c, d**). *P < 0.05. Error bars are mean ± SEM. Overlaid points are individual animal scores.

We next check the effect of increased spinal Rheb on mTORC1 activation in CCI model. CCI induces an obvious activation of spinal mTORC1 (Fig. 2). Increased phosphorylation of mTOR, S6 and 4-E-BP-1 was observed in the spinal cord of the mice in the CCI group (Fig. 2a–c, *P* < 0.05 as compared with the sham group). Immunofluorescence also showed that phosphorylation of spinal S6 was significantly increased in the dorsal horn in CCI group (Fig. 2d, *P* < 0.05, as compared with sham group).

### Blocking the mTORC1 signal with rapamycin could alleviate the development of neuropathic pain and restore morphine efficacy in CCI mice

**Fig. 3:**
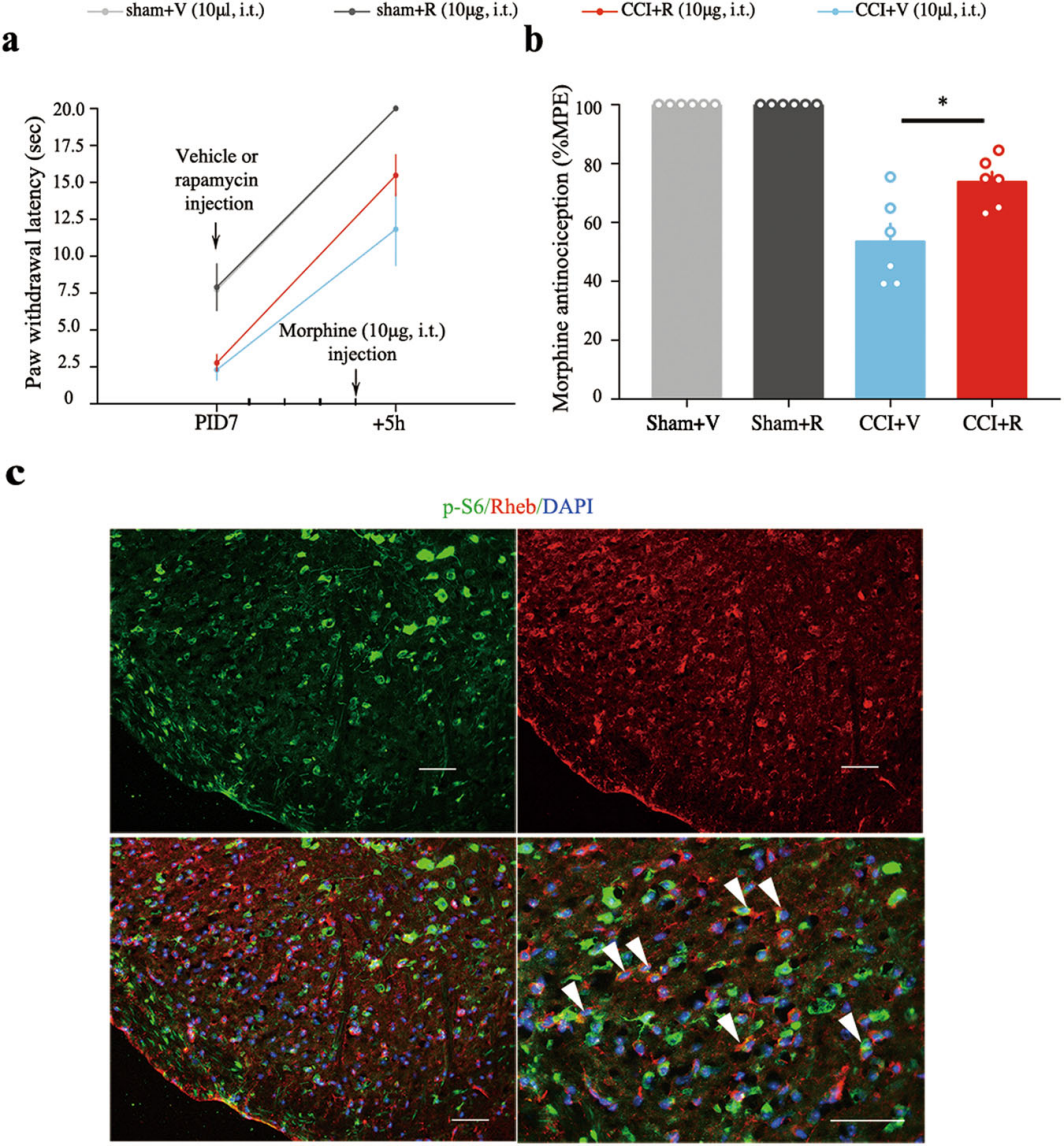
Bolus rapamycin restores aucte morphine efficacy in Neuropathic pain mice and CCI-induced Rheb co-localized with pS6 in the spinal dorsal horn. **a** Nociceptive behavior on day 7 after CCI injury and morphine antinociception while co-treatment with vehicle or rapamycin (*n* = 6); **b** intrathecal rapamycin (red) restores acute morphine efficacy in CCI model as compared with vehicle group (blue); **c** CCI-induced Rheb (red) co-localized with phosphorylated S6 (green) in the spinal dorsal horn in neuropathic pain mice. Repeated measures two-way ANOVA + Bonferroni (**b**). *P < 0.05. Error bars are mean ± SEM. Overlaid points are individual subject.

Intrathecally administrated an un-analgesic dose of rapamycin could effectively increase the thermal latency on day 3, 5, and 7 (Fig. 1a, b, *P* < 0.05 as compared with CCI with vehicle group). Bolus intrathecal rapamycin injection could effectively potentiate morphine-induced analgesic effect and increase the MPE% of morphine-induced antinociception 4 h after drug injection in established CCI model (Fig. 3a, b, *P* < 0.05 as compared with CCI with vehicle group). These findings suggest that neuropathic pain induced Rheb-mTORC1 activation might lead to a reduced morphine efficacy and increased tolerance tendency. Blockade of spinal mTORC1, not only restores morphine efficacy, but also could relieve the development of neuropathic pain.

### Upregulated Rheb was co-localized with the phosphorylated S6 in neurons of the spinal dorsal horn by double immunofluorescent staining in CCI model

Based on previous work, Rheb induced by inflammatory pain was expressed in both neurons and astrocytes^8^. To explore the role of Rheb-induced activation of mTORC1 in specialized cell types after CCI, double immunofluorescent labeling was employed. As shown in Fig. 2e, pS6 expressed predominantly in neurons (Fig. 2e, left panel), but not microglia (Fig. 2e, right panel) or astrocyte (Fig. 2e, middle panel). It was also shown that CCI-induced Rheb (red) co-localized with the phosphorylated S6 (green) in the spinal dorsal horn (Fig. 3c).

### Rheb S16H KI mouse mimic the phenotypes of impaired acute morphine efficacy and development of morphine-induced antinociceptive tolerance in CCI model

**Fig. 4:**
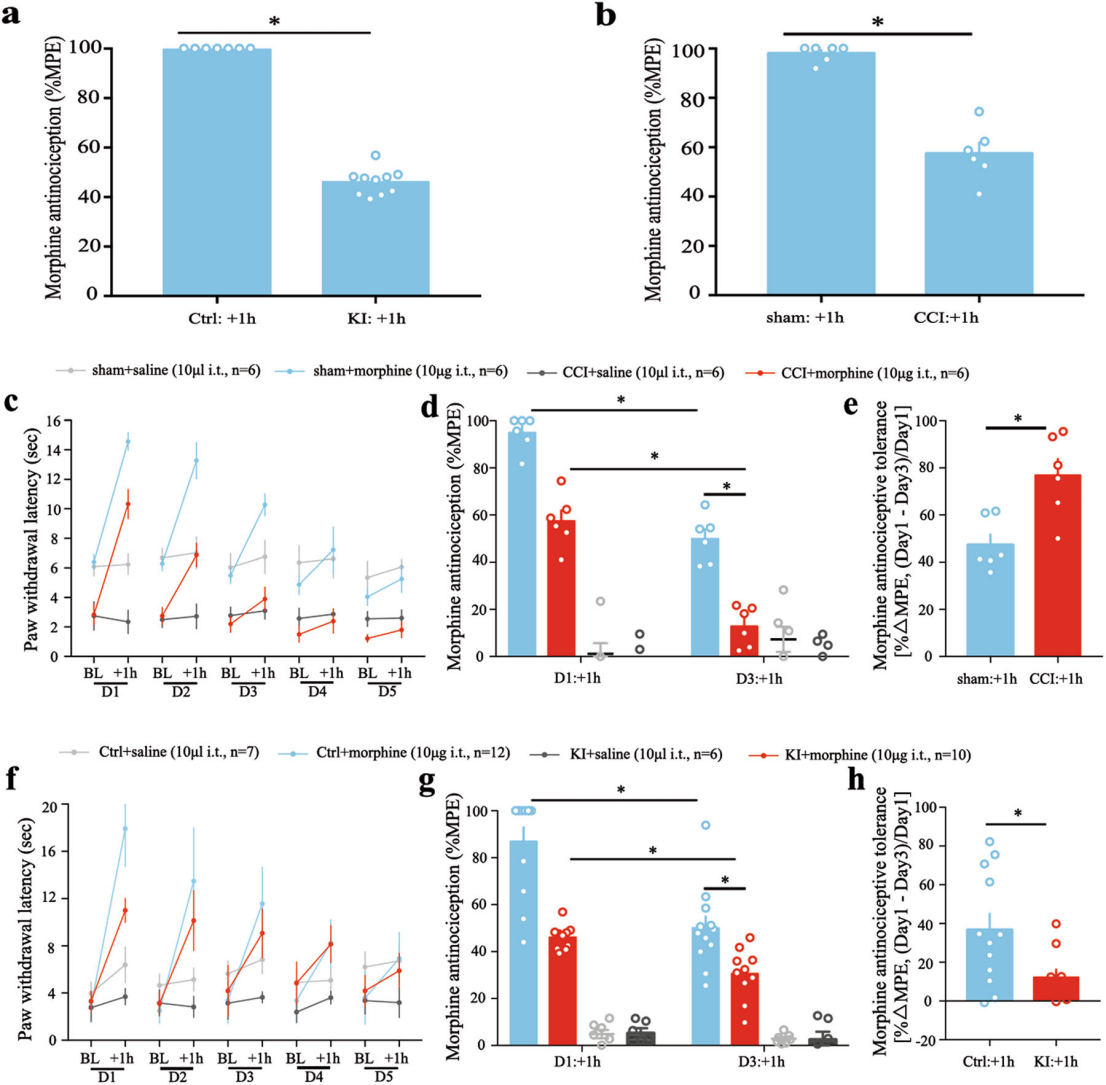
Rheb KI mice mimics the impaired acute morphine efficacy and increased tolerance phenotype of morphine-induced antinociception in CCI model. **a** Rheb knockin mice showed an impaired morphine-induced antinociception after acute intrathecal injection (10 µg, i.t.; *n* = 7 for littermate control group, *n* = 9 for CKI group); **b** antinociceptive efficacy of morphine (10 µg, i.t.) in mice with Chronic Constriction Injury (CCI) of the sciatic nerve after 7 days (*n* = 6 for sham group, *n* = 6 for CCI group); **c** Daily nociceptive behavior and opioid antinociception throughout a 5 day chronic morphine schedule (10 μg i.t., twice daily) in CCI mice; Nociceptive behavior (pre-morphine BL timepoints only): tail immersion; Antinociception (post-morphine 1 h timepoints only): tail immersion; antinociceptive tolerance: **d** maximal possible effect (MPE) for morphine antinociception from the first administration on day 1 (Day 1: +1 h) compared to the first administration on day 3 (Day 3: +1 h) (tail immersion), and **e** the percent change between day 1 and 3 of each subject. sham, *n* = 6; CCI, *n* = 6; **f** Daily nociceptive behavior and opioid antinociception throughout a 5 day chronic morphine schedule (10ug i.t., twice daily) in Rheb CKI mice and littermate control group; Nociceptive behavior (pre-morphine BL timepoints only): tail immersion; antinociception (post-morphine 1 h timepoints only): tail immersion; antinociceptive tolerance: **g** Maximal possible effect (MPE) for morphine antinociception from the first administration on day 1 (Day 1: +1 h) compared to the first administration on day 3 (Day 3: +1 h) (tail immersion), and **h** the percent change between day 1 and 3 of each subject. Littermate control group, *n* = 12; Rheb CKI, *n* = 10.

We measured thermal nociceptive latency before and 1 h after morphine injection of a Rheb S16H knockin mouse line^9^. Acute intrathecal morphine-induced antinociception was significantly impaired as compared with the littermate control mice (Fig. 4a, *P* < 0.05). Acute morphine-induced %MPE was impaired and just could reach 60% in the mice of CCI group as compared with sham group (Fig. 4b, *P* < 0.05). And the phenotype of impaired morphine effect in Rheb S16H knockin mouse looks like the morphine performance in CCI model (Fig. 4b).

Repeated morphine treatment promoted the development of morphine-induced antinociceptive tolerance on day 3 in neuropathic pain mouse (Fig. 4c, d), while morphine still could induce significant antinociception in sham mice (Fig. 4c, d, *P* < 0.05 as compared sham mice receive saline group). The delta MPE% of morphine-induced antinociception between day 1 and 3 was greater in CCI group than that in sham group (Fig. 4e, *P* < 0.05). The development of morphine-induced antinociceptive tolerance showed a similar behavioral phenotype in Rheb KI mice (Fig. 4f–h, *P* < 0.05) as compared littermate control group receive morphine treatment.

Similar impaired morphine-induced antinociceptive effects and tolerance phenotypes in Rheb S16H knockin mice and neuropathic pain models imply a potential shared mechanism such as persistent Rheb-induced spinal hypersensitivity by these two models.

### Inhibition of mTORC1 with its antagonist, rapamycin, reverses the increased spinal neuronal activity in morphine-induced tolerance and neuropathic pain models

**Fig. 5:**
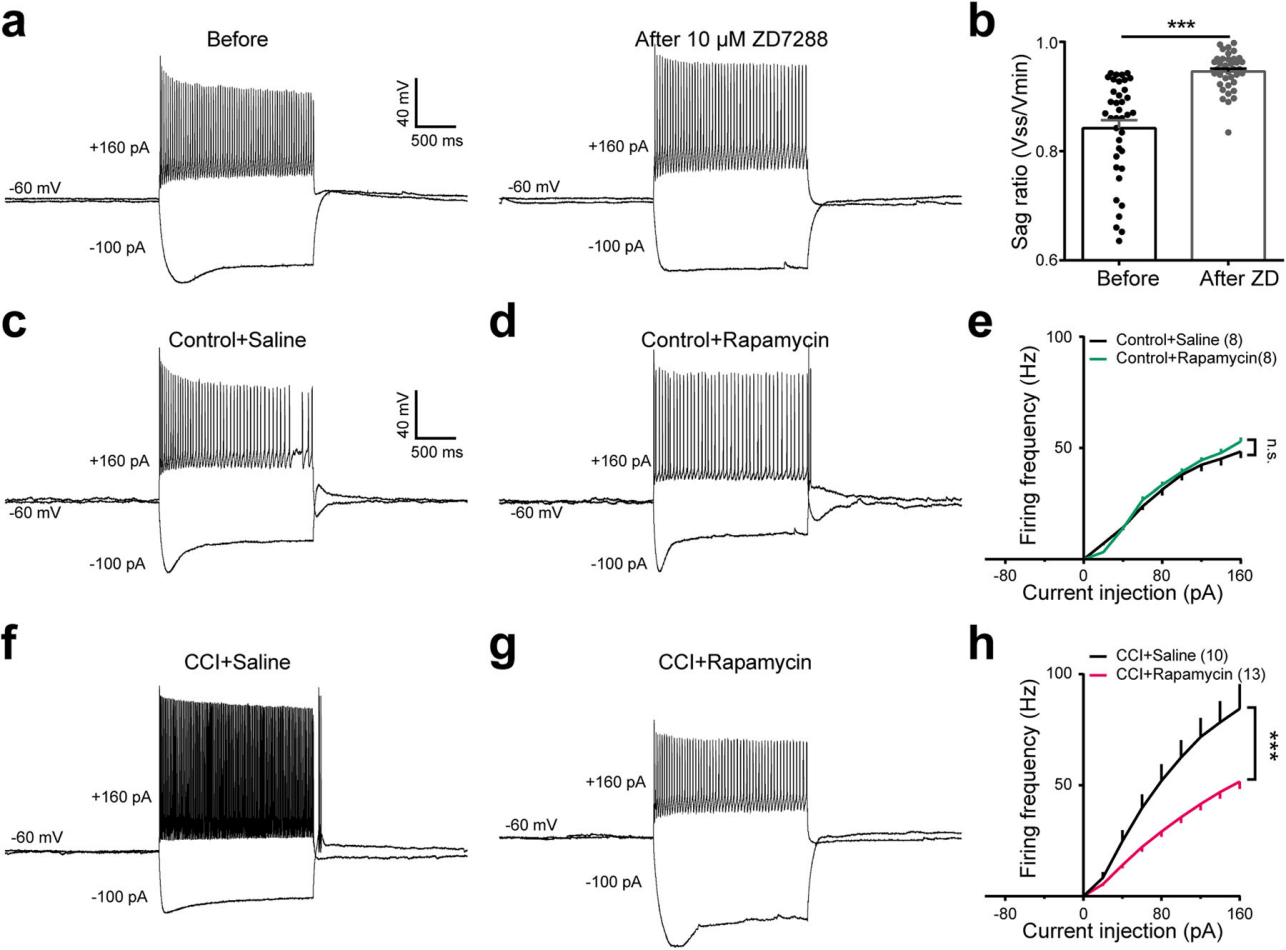
Rapamycin suppressed the dorsal horn neuron’s spike firing in morphine-induced tolerance and neuropathic pain models. **a** Representative traces showing dorsal horn neuron’s voltage responses evoked by current injections (−100 pA, 160 pA) before (left) and immediately after (right) bath application of 10 μM ZD7288, *n* = 40; **b** Sag ratio of the same neuron before and after bath application of 10 μM ZD7288; representative traces showing dorsal horn neuron’s voltage responses evoked by current injections (−100 pA, 160 pA) in control mice after 5 day continuously application of saline (**c**) or rapamycin (**d**), summary of data showing the effect of current injection evoked spike firing after application of saline or rapamycin (**e**), *n* = 8. Representative traces showing dorsal horn neuron’s voltage responses evoked by current injections (−100 pA, 160 pA) in CCI induced neuropathic pain model after 5 day continuously application of saline (**f**) or rapamycin (**g**), summary of data showing the effect of current injection evoked spike firing after application of saline or rapamycin (**h**), *n* = 10 in CCI + saline and 13 in CCI + rapamycin. The paired Student’s *t* test was performed for the data in (**b**) and Kolmogorov–Smirnov test for the data in (**e**, **h**). n.s., not significant; *P < 0.05, ***P < 0.001. Data are represented as mean ± SEM.

Neuropathic pain produces significant hyperalgesia, which is characterized by hyperexcitability of spinal cord neurons^10,11^. The firing patterns of dorsal horn neurons are extremely heterogeneous, we need to select a special type neurons for study^12^. Increasing evidence has indicated that hyperpolarization active cyclic nucleotide (HCN) channels expressed in spinal cord were involved in pain generation and maintenance^13–15^. To investigate the neural excitability before and after inhibition of mTORC1 with rapamycin, we performed whole-cell patch clamp recording in laminae II dorsal horn neurons^12^. To determine the neurons we recorded were expressed HCN channels, 10 μM ZD7288 (antagonist of HCN channel) was applied to the same cell (Fig. 5a, b). After 5 consecutive days application of rapamycin, dorsal horn neurons in wild-type mice have no significant difference with saline injection group (Fig. 5c–e; *P* = 0.9). However, in CCI induced neuropathic pain model, dorsal horn neurons exhibit apparent hyperexcitability (Fig. 5f). After 5 consecutive day injection of rapamycin, the current injection evoked firing of dorsal horn neurons was significantly suppressed (Fig. 5g, h; *P* < 0.001).

## Discussion

mTOR pathway governs a variety of neuronal functions, including cell proliferation, survival, growth, and plasticity. Increased mTOR signaling has shown to provoke neuronal hyperexcitability^16,17^, which maybe the nature of neuropathic pain^18^. Protein translations triggered by mTORC1 signaling are necessary for the initiation and maintenance of chronic pain^19^. Here, we show that neuropathic pain could induce persistent Rheb-mediated mTORC1 activation and spinal hyperexcitability. And the genetic Rheb knockin mouse showed an upregulated activation of Rheb-induced mTORC1 and mimics the behavioral characteristics of this disease model. Neuropathic pain induced spinal hyperexcitability in dorsal horn neurons could be blocked by mTORC1 inhibitor, rapamycin.

Rheb is vital in regulation of growth and cell cycle progression due to its role in the insulin/TOR/S6K signaling pathway and is reported to activate mTORC1^20^ and positively regulate mTOR activation^21^. The rapamycin-sensitive mTORC1 complex is predominantly responsible for regulating protein translation and is formed with active mTOR bound to Raptor protein. The detailed mechanisms of neuropathic pain are not fully illustrated, but are generally proposed as adaptations within the peripheral and CNS. Adaptive changes often result from changes in protein translation governed by mTORC1 signaling^22^. Rheb mediated activation of mTORC1 may lead to the spinal neuronal sensitization induced by pain or morphine antinociceptive tolerance as described above. In this study, we demonstrated a significant increase of spinal Rheb in CCI-induced neuropathic pain and we provided direct evidence through the genetic knockin neuronal Rheb mouse model that persistent Rheb-induced mTORC1 activation mediates spinal sensitization which might be an underlying mechanism of the clinical pathological state.

Rheb and mTOR have been proposed to participate in chronic pain process. Previous researchers showed that spinal Rheb mRNA was time-dependently increased following carrageenan injection and thought that spinal mTORC1 regulate the nociception both in the peripheral and the CNS^21^. Inhibition of mTOR activity by rapamycin was reported to block hypersensitivity evoked by inflammatory pain, or neuropathic pain^23,24^ and attenuate formalin, capsaicin and nerve ligation-induced nociceptive behaviors^24,25^. mTOR activation results in phosphorylation of 4E-BP1, S6, and S6K which may correlate with the initiation of hypersensitivity and spinal sensitization^21^ as well as persistent pain states. Increased phosphorylation of S6 and 4-E-BP1 was also reported to be decreased by centrally application of rapamycin^25^. Geranton et al. declared that rapamycin-sensitive signaling pathway is necessary for the full expression of persistent pain states. CCI was reported to induce pain hypersensitivity through activation of spinal mTOR in mice, not only CCI induced upregulated phosphorylation of mTOR, 4E-BP1, and S6K, but also mechanical allodynia were attenuated by intrathecal injection of rapamycin^26^. Here, we proved that CCI induced an obvious increase of spinal Rheb expression which stimulates the activation of mTORC1 in the spinal dorsal horn. Application of rapamycin not only potentiates the antinociceptive efficacy of morphine, but also partially prevent the development of thermal hyperalgesia in neuropathic mouse model. In addition, rapamycin could even reverse CCI-induced thermal hyperalgesia after acute intrathecal application. The behavioral tests demonstrated that morphine-induced antinociceptive efficacy was facilitated in CCI neuropathic pain model. Furthermore, CCI-induced S6 phosphorylation mainly expressed in neurons and co-localized with Rheb in spinal dorsal horn. All of these imply a pivotal role of spinal Rheb-induced mTORC1 activation in the induction and maintenance of neuropathic pain.

The same as Rheb-induced mTORC1 activation in neuro-inflammation^8^, neuropathic pain induced phosphorylation of S6 also co-localized with neuron, but not with astrocyte or microglia. As dorsal horn is strongly implicated in pain modulation, neuronal Rheb-induced persistent activation of mTORC1 may play a vital role in the increased neuroplasticity of chronic pain. Neuroplasticity in the form of adaptive changes in protein transcription and translation may contribute to the induction and maintenance of chronic pain^7^. mTOR-mediated translational control is critical for synaptic plasticity through regulation of protein synthesis^27^. Rheb also has been proposed to regulate mTOR-dependent protein translation in neuronal dendrites and modulate synaptic plasticity^28^. Yamagata et al. suggested Rheb as a synaptic activity-regulated gene and play an vital role in long-term activity-dependent neuronal responses^29^. Further more, neuropathic pain-induced phosphorylation of S6 mainly located in the excitatory neurons which corresponds to pain-related synaptic plasticity in dorsal horn of spinal cord^30^. Rheb-induced synaptic plasticity may lead to increased neuronal excitability in the spinal cord and accelerate the development of neuropathic pain.

Peripheral signals of injury pain generate increased neuronal excitability in the spinal cord^31^. HCN ion channels were reported to play a central role in inflammatory and neuropathic pain^13^. Here, we selected the neurons expressed HCN currents in the dorsal horn to record their electrophysiological activity and proved that the firing rate was typically increased in the spinal neurons after CCI model established. Interestingly, this increased neuronal excitability could be alleviated when rapamycin was applied. Previous work reported that mTORC1 might participate in activity-dependent increase of excitability^32^, and Rheb-induced mTOR hyperactivation was reported to cause a constellation of neurodevelopmental disorders accompanied by hyperexcitable cortical malformations and colocalize with hyperexcitability^33^. Taken together, these evidences show that neuropathic pain may induce increased spinal hypersensitivity, which is mediated by Rheb-induced mTORC1 activation, and lead to spinal sensitization.

PI3K signaling was reported to regulate expression of Rheb previously^34,35^ and the intracellular PI3K/Akt mediated activation of spinal mTORC1 in morphine-induced tolerance and hyperalgesia^36^. Recent work showed that PI3K/Akt signal is involved in the induction and maintenance of neuropathic pain^37^ and spinal central sensitization of neuropathic pain^38^. Although PI3K/Akt signaling may originate from different mechanisms in neuropathic pain, the downstream target Rheb-mTORC1 maybe an underlying pathway to induce spinal sensitization.

In conclusion, this study indicates that spinal Rheb-mTOR signal paly an important role in regulation of spinal sensitization in neuropathic pain, and suggests that targeting mTOR pathway may provide a new strategy for pain management.

## Materials and methods

### Animals

Adult C57bl6 male mice weighing 20–25 g were housed in groups of five in cages (32 × 21 × 16 cm) on corn cob. Keep the feeding temperature at 22–23 °C with an alternating 12-h light/dark cycle (lights on at 07:00 h). Water and food were available ad libitum.

### Ethics approval

The protocol followed the NIH Guide for the Care and Use of Laboratory Animals (NIH Publications No. 8023, revised 1978), and was approved by the Animal Ethics Committee of the Sixth People’s Hospital Affiliated to Shanghai Jiao Tong University, and are reported in accordance with the ARRIVE guidelines. Efforts were made to minimize suffering and reduce the number of animals used.

### Rheb1 S16H transgenic conditional knockin mice

Mice with nestin Cre and Rheb1 S16H knockin allele were generated in Worley lab^9^. Nestin Cre transgenic mice were crossed with floxed Rheb1 S16H mice to generate cKI line. The genotype was determined by PCR with the following primers: WTF1 (forward): 5′-GCA CTT GCT CTC CCA AAG TC-3′; WTR1 (reverse): 5′-GCG GGA GAA ATG GAT ATG AA-3′) to amplify wt allele (596 bp); FloxF (forward): 5′-GCA ACG TGC TGG TTA TTG TG-3′; FloxR(reverse): 5′-GGG GAA CTT CCT GAC TAG GG-3′ to amplify the knockin allele (395 bp). The primers for the amplication of nestin Cre are 5′-TGC CAC GAC CAA GTG ACA GCA ATG -3′, and 5′-ACC AGA GAC GGA AAT CCA TGG CTC-3′ with the amplicon of 400 bp.

### CCI model

The CCI model was established under deep anesthesia as described before^39^. Animals were then randomized to CCI or sham group. In sham group, the surgical procedure was the same, but the nerve was not ligated. During the operation, the concentration of the inhaled anesthetic was adjusted when necessary.

### Behavioral testing

#### Paw withdrawal latency

The plantar stimulator analgesia meter (IITC Inc./Life Science Instruments, Woodland Hills, CA) was used to measure thermal paw withdrawal latency (PWL). Briefly, radiant heat extends from the bottom to the middle of the plantar of the ipsilateral hind paw. The time between the light onset and the foot lift is defined as the PWL. Each test was repeated three times at a 10-min interval to prevent sensitization. A 20-s cutoff time was set to avoid tissue damage.

#### Tail-flick latency

Pain response to thermal stimulation was evaluated with warm water (52.5 ± 0.5 °C) immersion^40,41^. In short, dip the tip of the mouse’s tail (about 2 cm) into the warm water, and record the time taken to escape. To avoid injury, a 10-s cutoff was set. Response latency was recorded three times, with a 10-min interval between each read.

### Drug application

Morphine (Shenyang First Pharmaceutical Factory, Shenyang, China) was dissolved in saline at a final concentration of 1 µg/µl. Rapamycin (Sigma Aldrich, St. Louis, USA) was dissolved in DMSO (0.1%) in saline at a final concentration of 1 µg/µl. The drugs were delivered using an insulin syringe (BD, USA) into the subarachnoid^42^.

### Experimental design

We first examined the time course of behavior changes after establishing the CCI model. The PWL were examined for ipsilateral hind paws in CCI and sham group. The expression of Rheb and mTORC1 were detected by western blot, and the cell type of mTORC1 activation by CCI was observed by immunouorescence. To determine the effect of rapamycin in CCI mice, we examined the behavioral changes of the four groups: sham with vehicle, sham with rapamycin, CCI with vehicle and CCI with rapamycin. To determine whether rapamycin reversed thermo hyperanalgesia induced by CCI, vehicle or rapamycin was injected on day 7 and behavioral changes were examined in two groups 4 and 24 h later. To determine the analgesia of morphine on CCI, behavioral changes were examined in four groups every day: sham with saline, sham with morphine, CCI with saline, CCI with morphine. To determine whether rapamycin potentiated the analgesia of morphine on CCI, vehicle or rapamycin was injected 4 h before morphine injection on day 7 and behavioral changes were examined in four groups before and 60 min after morphine injection: sham with vehicle, sham with rapamycin, CCI with vehicle and CCI with rapamycin.

In the second part, we evaluated the analgesia effect of morphine in KI mice, behavioral changes were examined in four groups: control with saline, control with morphine, KI with saline, KI with morphine. Tolerance was established by intrathecally micro-injection of morphine twice per day for consecutive 5 days.

### Western blotting

Under deep anesthesia, the lumbar spinal cord were collected and immediately homogenized in tissue protein extraction reagent. Samples were prepared as reported previously^43^. Membranes were incubated with the following primary antibodies: mouse anti-actin (CST, MA, USA), mouse anti-Rheb (Santa Cruz, CA, USA), rabbit anti-p-mTOR (CST, MA, USA), rabbit anti-mTOR (CST, MA, USA), rabbit anti-pS6 (CST, MA, USA), rabbit anti-S6 (CST, MA, USA), rabbit anti-p4E-BP1 (CST, MA, USA), rabbit anti-4E-BP1 (CST, MA, USA). The blots were washed in TBST and then incubated with secondary antibody (Huaan Biotechnology, Hangzhou, China). Bands were detected by Image Quant Ai600 (General Electric Co., USA) with ECL substrate (Thermo Fisher). The results were analyzed and quantified using ImageJ software (version 2.0.0, USA).

### Immunofluorescence

For immunofluorescence, mice were anaesthetized and transcardially perfused with 4% cold paraformaldehyde. The lumbar spinal cord was harvested, and then dehydrated in 10, 20, and 30% sucrose sequentially. In CCI group, co-localization of pS6 or Rheb with NeuN (a marker of neuron), CD11b (a marker of microglia), GFAP (a marker of astrocyte) and pS6 with Rheb were assessed by double-labeling. Slides were incubated with the primary antibody (mouse anti-Rheb, Santa Cruz Biotechnology; rabbit anti-pS6, mouse anti-NeuN, Thermo fisher; mouse anti-CD11b, Abcam; mouse anti-GFAP, Abcam) overnight at 4 °C^43^. The slides were then rinsed in PBS and then incubated with the secondary antibodies (goat anti-rabbit IgG H&L, Alexa Fluor^®^ 594 and 488, and goat anti-mouse IgG H&L, Alexa Fluor^®^ 594 and 488, Abcam) for 2 h at room temperature. Images were acquired with a fluorescence microscope (Leica DM IL LED, Buffalo Grove, IL, USA).

### Electrophysiological recording

Spinal cord slices were prepared as described previously^44^. Mice (P15-P25) were deeply anesthetized. The spinal cord were quickly excised and placed in ice cold cutting solution containing (in mM): sucrose 50, KCl 1.8, NaCl 95, KH_2_PO_4_ 1.2, MgSO_4_ 7, CaCl_2_ 0.5, NaHCO_3_ 26, glucose 15 with pH at 7.4 and osmolarity at 310–320 mOsm, and oxygenated with 95% O_2_ and 5% CO_2_. Transverse slice (300 μm) were cut from the lumbar spinal cord by a vibratome VT1200 (Leica, Germany). Slices were then incubated for 40 min at 34 °C ACSF containing (in mM): NaCl 127, KCl 1.8, KH_2_PO_4_ 1.2, MgSO_4_ 1.3, CaCl_2_ 2.4, NaHCO_3_ 26, glucose 15 and bubbled with 95% O_2_ and 5% CO_2_ (310–320 mOsm). The slice was then transferred to a recording chamber and continuously perfused with oxygenated ACSF at a rate of 3 ml/min at room temperature (22–26 °C).

Whole-cell patch-clamp recordings were acquired with an EPC-10 triple amplifier (HEKA, German), and signals were filtered at 2.9 kHz and sampled at 15 kHz. The recording micropipettes were made from borosilicate glass capillaries (Sutter, USA) with a resistance of 5–8 MΩ. The internal solution contained (in mM): K-gluconate 125, Na_2_ATP 3, NaGTP 0.5, MgCl_2_ 2, CaCl_2_ 2, HEPES 10, EGTA 10 (PH 7.5). Neurons were randomly picked in laminae II of dorsal horn. Sag ratio was calculated by dividing member voltage at steady state (Vss) by the minimum membrane voltage after current injuction (Vmin). ZD7288 diluted in the ACSF and perfused at a speed about 3 ml/min.

### Statistical analysis

The sample size of each group in this study was determined based on previous report^45^. Datas of PWL and TFL were expressed as %MPE = 100 × (postdrug latency threshold – predrug latency threshold)/(cutoff latency threshold – predrug latency threshold) and were analyzed by two-way ANOVA followed by one-way ANOVA and Tukey’s test for multiple comparisons. For western blot and immunofluorescence, relative expression of target proteins in different groups was normalized to β-actin, and phosphorylation level of target proteins was compared with their total level. The distribution of the data was assessed by Kolmogorov–Smirnov test at first. If data follows Gaussian distribution, student *t* test was carried out. Post hoc tests were run only when F achieved *P* < 0.05. If data was not normally distributed, nonparametric tests were used. Differences were considered significant at *P* < 0.05. All data analysis were performed by observers blinded to the experimental groups and were presented as the mean ± SD and analyzed with Graph-Pad Prism 5 (San Diego, CA, USA). No randomization was used. No blinding was done.

## Supplementary information

Supplemental Data

Figure S1

## Data Availability

All data generated or analyzed during this study are included in this published article and its supplementary information files.
