# Supplementary material for: Persistent Rheb-induced mTORC1 activation in spinal cord neurons induces hypersensitivity in neuropathic pain
Source: Cell Death Dis. 2020 Sep 12;11(9):747. doi: 10.1038/s41419-020-02966-0 (PMC7487067; doi:10.1038/s41419-020-02966-0)
Supplement: Supplementary file 1 — Supplemental Data [file 41419_2020_2966_MOESM1_ESM.docx]

**Supplement 1: Western blot analysis of spinal cord to assess expression of Rheb and activation of mTORC1 signal in Rheb KI mice and little mate control mice.**

(a) Spinal Rheb was significantly increased in the Rheb S16H mice (p < 0.05 as compared with litter mate control); (b) Phosphorylation of mTOR was significantly increased in the Rheb S16H mice (p < 0.05 as compared with litter mate control); (c) Phosphorylation of S6 was significantly increased in the Rheb S16H mice (p < 0.05 as compared with litter mate control); (d) Phosphorylation of 4-E-BP1 was significantly increased in the Rheb S16H mice (p < 0.05 as compared with litter mate control). Student’s t Test, two-tailed (a, b, c and d) *P < 0.05. Error bars are mean ± SEM. Overlaid points are individual animal scores.
