# Supplementary figures and images for: Persistent Rheb-induced mTORC1 activation in spinal cord neurons induces hypersensitivity in neuropathic pain
Source: Cell Death Dis. 2020 Sep 12;11(9):747. doi: 10.1038/s41419-020-02966-0 (PMC7487067; doi:10.1038/s41419-020-02966-0)

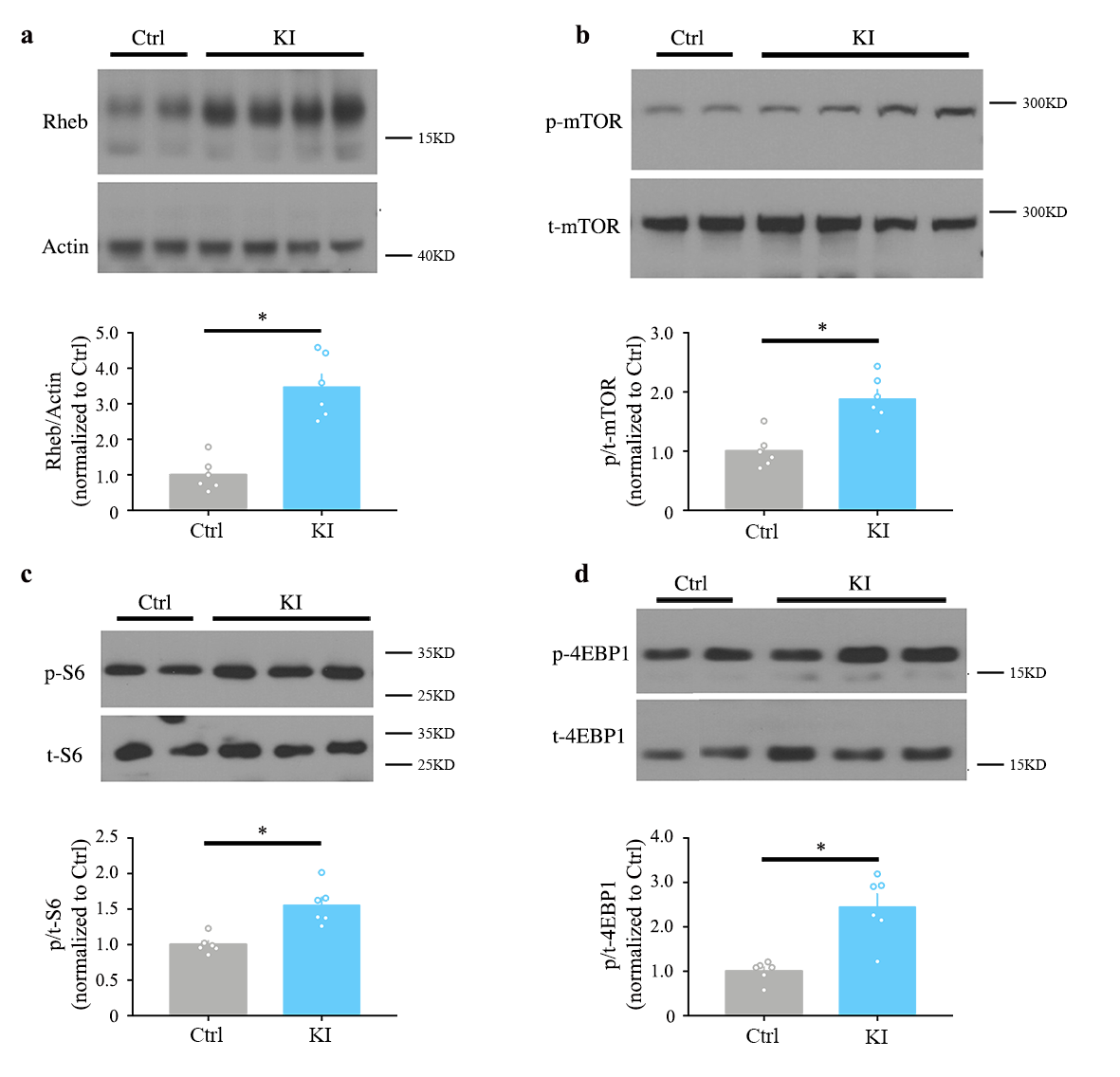

Supplement: Supplementary file 2 — Figure S1 [file 41419_2020_2966_MOESM2_ESM.tif]
